# A genome-wide scan for diversifying selection signatures in selected horse breeds

**DOI:** 10.1371/journal.pone.0210751

**Published:** 2019-01-30

**Authors:** Artur Gurgul, Igor Jasielczuk, Ewelina Semik-Gurgul, Klaudia Pawlina-Tyszko, Monika Stefaniuk-Szmukier, Tomasz Szmatoła, Grażyna Polak, Iwona Tomczyk-Wrona, Monika Bugno-Poniewierska

**Affiliations:** 1 Department of Animal Molecular Biology, National Research Institute of Animal Production, Balice, Poland; 2 Department of Horse Breeding, University of Agriculture in Kraków, al. Kraków, Poland; 3 Department of Horse Breeding, National Research Institute of Animal Production, Balice, Poland; 4 Institute of Veterinary Sciences, University of Agriculture in Krakow, al. Kraków, Poland; University of Illinois, UNITED STATES

## Abstract

The genetic differentiation of the current horse population was evolutionarily created by natural or artificial selection which shaped the genomes of individual breeds in several unique ways. The availability of high throughput genotyping methods created the opportunity to study this genetic variation on a genome-wide level allowing detection of genome regions divergently selected between separate breeds as well as among different horse types sharing similar phenotypic features. In this study, we used the population differentiation index (F_ST_) that is generally used for measuring locus-specific allele frequencies variation between populations, to detect selection signatures among six horse breeds maintained in Poland. These breeds can be classified into three major categories, including light, draft and primitive horses, selected mainly in terms of type (utility), exterior, performance, size, coat color and appearance. The analysis of the most pronounced selection signals found in this study allowed us to detect several genomic regions and genes connected with processes potentially important for breed phenotypic differentiation and associated with energy homeostasis during physical effort, heart functioning, fertility, disease resistance and motor coordination. Our results also confirmed previously described association of loci on ECA3 (spanning *LCORL* and *NCAPG* genes) and ECA11 (spanning *LASP1* gene) with the regulation of body size in our draft and primitive (small size) horses. The efficiency of the applied F_ST_-based approach was also confirmed by the identification of a robust selection signal in the blue dun colored Polish Konik horses at the locus of *TBX3* gene, which was previously shown to be responsible for dun coat color dilution in other horse breeds. F_ST_-based method showed to be efficient in detection of diversifying selection signatures in the analyzed horse breeds. Especially pronounced signals were observed at the loci responsible for fixed breed-specific features. Several candidate genes under selection were proposed in this study for traits selected in separate breeds and horse types, however, further functional and comparative studies are needed to confirm and explain their effect on the observed genetic diversity of the horse breeds.

## Introduction

The present horse population abounds in the variety of phenotypes resulting mainly from selective breeding directed at improvement of particular phenotypic features. Since the domestication, various selection criteria aimed towards the improvement of horse usability in transportation, agriculture or horsemanship have been applied [1–2]. This drove the specialization of particular populations in terms of utility and finally resulted in a creation of formal breeds constituting largely closed populations with high genetic uniformity of individuals within breeds [3]. The current selection in the most of horse breeds is primarily directed at the improvement of traits connected with appearance and performance. However, apart from the highly specialized breeds, there are also horse populations which are valued for their primitive nature manifesting in their robust constitution for primitive living conditions [4]. These horse populations were mainly shaped by natural selection, however, selective breeding for breed standard and mating in closed populations makes their genetic characteristics largely similar to that found in other horse breeds.

Both natural and artificial selection lead to changes in allele frequencies between populations, and over time, different variants and haplotype structures are being fixed within separate breeds [5]. Various concepts and statistics have been used to detect such selection signatures from genome-wide SNP data in livestock species. Some of them are based on within breed genome characteristics and other relay on genetic variation among breeds. Most of the available methods are based on: (i) the high frequency of derived alleles and other consequences of hitchhiking within population, (ii) the length and structure of haplotypes, measured by extended haplotype homozygosity (EHH) or EHH-derived statistics, and (iii) the genetic differentiation between populations, measured by F_ST_ or related statistics [6]. The genetic differences arising as a result of selection are presumably concentrated at the functional variants loci being beneficial for the selected traits. Thanks to the linkage disequilibrium across the genome, the approximate position of these functional variants can be identified using neutral genome-wide SNP panels and a comparative analysis of allele frequency distribution between different breeds [5]. A commonly used approach to detect diversifying selection signatures is based on the measure of population differentiation due to locus-specific allele frequencies variation between populations, which is quantified using the F_ST_ statistic [7] and further averaged at specific chromosome distances to account for stochasticity [8]. F_ST_ provides information on the genomic variation at a locus among populations relative to that found within populations [9]. These among-breed differences, which can be considered as diversifying selection signals, have been previously successfully used to map genomic loci spanning the genes with variants responsible for a number of phenotypic features in different species, including coat color, size, muscling, production and reproduction [10–14]. The identification of selection signals across the genome connected with candidate gene approach was also useful to detect loci associated with size, performance, coat color and gait in several horse breeds [2,4,15,16].

To extend the knowledge in this area and provide more data on candidate gene loci responsible for important breed specific features, in this study, we attempt to identify selection signatures by the analysis of genetic diversity of six different horse breeds which can be classified into three major categories: light, draft and small size primitive horses. Among the light horses, apart from well-known Arabian horses we analyzed Małopolski horse which is currently well-balanced riding horse that was originally developed mainly from native Polish population crossed with Thoroughbreds and Arabians. The primitive breeds analyzed in this study included Hucul and Polish Konik. Both these breeds are represented by a small size horses with several characteristics of feral population. Hucul horses originate from Carpathian Mountains and most likely, they are descendants of various types of horses: Tatar, Oriental, Arabian, Turkish, Przewalski’s horses, as well as horses with Norse blood. The Polish Konik originate from a now-extinct Tarpan horse from native Polish population and is characterized by several primitive features, including mouse-dun coat color and a dorsal stripe. The draft horses analyzed in this study included Sokolski and Sztumski breeds. Sokolski horse derives from cross-breeding of local Polish mares of Polish Coldblood type with imported Ardennais and Breton sires. The Sztumski horse is the largest and the heaviest of cold-blooded horses maintained in Poland which was originally created on the basis of local population crossbred mainly with Ardennes and Belgian sires [17]. The direct comparison between these horse types and breeds can provide candidate gene loci presumably responsible for constitution, size and coat color and may also contribute to the recognition of the genetic background and source of variation of horse phenotypic features. Five out of six breeds analyzed in this study are included in the conservation programs following FAO National Rare Livestock Breeds Preservation guidelines and additionally, according to our best knowledge, these native horse breeds were not studied before in terms of diversifying selection and thus this study provides new data on horse breeds genetic diversity and variation.

## Methods

### Animals, samples and genotyping

The study material comprised blood samples obtained from 571 horses (both males and females) randomly sampled from different herds and belonging to six different breeds. The breeds were selected to represent three major horse types: light horses–Arabians (*n* = 124; AR) and Malopolski horse (*n* = 56; MLP); primitive horses–Hucul (*n* = 116; HC) and Polish Konik (*n* = 99; KN) breeds; and draft horses–Sokolski (*n* = 107; SOK) and Sztumski (*n* = 69; SZTUM). The blood was collected from jugular vein by a veterinary doctor in an amount of 10 ml to a vial containing EDTA K3. Samples from Arabian horses were obtained from three different studs: SK Janów (Lubelskie province), SK Michałów (Świętokrzyskie Province) and SK Białka (Lubelskie province) which were our project partners. In the case of Małopolski horses–the material was sampled in all above studs and in farms of individual breeders with their personal consent. The biological material of Hucul horses was obtained in the Gładyszów Stud (Małopolskie province) and ZDIZ PIB Odrzechowa (Podkarpackie province) with the consent of their Chairmans. In the case of Polish Konik, the material was sampled in: Popielno Research Station, IRiŻZ PAN (Warmian-Masurian province) and Kalitnik—PTOP Research Station (Podlasie province) with the consent of the Presidents. The biological material from draft horses was collected in herds participating in the programs for the conservation of genetic resources of Sztumski and Sokolski horses. The herds were located in the Podlasie (Sokólski) and Pomorskie (Sztumski) provinces. Each farmer participating in the program signed a cooperation agreement with the NRIAP, where they undertake to provide data and biological material for research purposes. Breeders participating in the conservation program are obliged to keep animals in accordance with the criteria of animal welfare, which is a subject to the control of district veterinarians. The genomic DNA was purified from the blood using a Sherlock AX kit (A&A Biotechnology) and after a quality control was genotyped with the use of the Neogen Equine Community BeadChip assay (Illumina) according to the standard Infinium Ultra protocol. All animal procedures were approved by the Local Animal Care Ethics Committee No. II in Kraków–permission number 1293/2016 in accordance with EU regulations.

### Data filtering

All sampled animals were genotyped with the use of Neogen Equine Community array (Illumina) including probes for 65,157 SNPs with an average inter-marker distance of 36.3 kb. Only genotypes with call rate >0.97 were used for the analysis. The initial SNPs set was filtered to remove markers located on the sex chromosomes (EquCab2.0 genome build). The initially filtered SNP panel included 61,268 markers, which was further reduced by applying population-wide filters. The filters included MAF threshold of 5% and <20% of missing genotypes in the whole studied population. Additionally, SNPs with critical p-value for HWE <1.0E-06 in each breed separately were excluded. This resulted in the final SNP panel of 52,023 markers, scattered across the genome with an average inter-marker distance of 43.0 kb.

### Identification of diversifying selection signatures

The genome regions with differentially fixed variants or strongly differing in alleles frequency between separate breeds were identified using pairwise Wright’s F_ST_ [9], the conventional measure of population genetic differentiation. The F_ST_ values obtained for pairwise comparisons at each SNP were breed-standardized according to a methodology proposed by Akey *et al*. 8]. The standardized F_ST_ values were calculated (d_i_) as:

$$d_{i}=\sum_{j\neq i}\frac{F_{ST}^{ij}-E[F_{ST}^{ij}]}{sd[F_{ST}^{ij}]}$$

where $E[F_{ST}^{ij}]$ is expected value and $sd[F_{ST}^{ij}]$ denotes the standard deviation of F_ST_ between breeds *i* and *j* calculated from all analyzed SNPs. To account for random locus-by-locus variation, a 10-SNP sliding window was applied on the obtained d_i_ values. Candidate regions affected by diversifying selection were defined as the 99.9th percentile of the empirical distributions of window-averaged d_i_ values. The overlapping regions under selection were merged and (while searching for potentially linked genes) regions were extended on both ends by 25kb. Additional comparisons using d_i_ values were made between major horse types (light, primitive and draft) to detect potential selection differences between them. The linkage disequilibrium (LD) and haplotype block structure at the most diversified regions among the studied breeds were analyzed using HaploView 4.2 [18] software examining pairwise LD on the distance up to 500kb and detecting blocks based on a method proposed by Gabriel *et al*. [19]. Additionally, a detailed analysis was performed for previously detected candidate genes loci for horse body size (*LCORL/NCAPG*) [2,20] and the dun coat color phenotype (*TBX3*) [21].

Populations differentiation visualization was performed using principal component analysis on SNP genotypes and cladogram created based on weighted F_ST_ distances using neighbor-joining method [22].

The functional annotation of genes detected within the strongest selection signals was performed using the KOBAS 3.0 web server [23] and Panther Classification System [24]. A gene list enrichment analysis was done according to all known horse genes (genome) applying a correction for multiple testing.

## Results

### SNP panel statistics and general genetic differentiation of the studied breeds

**Fig 1:**
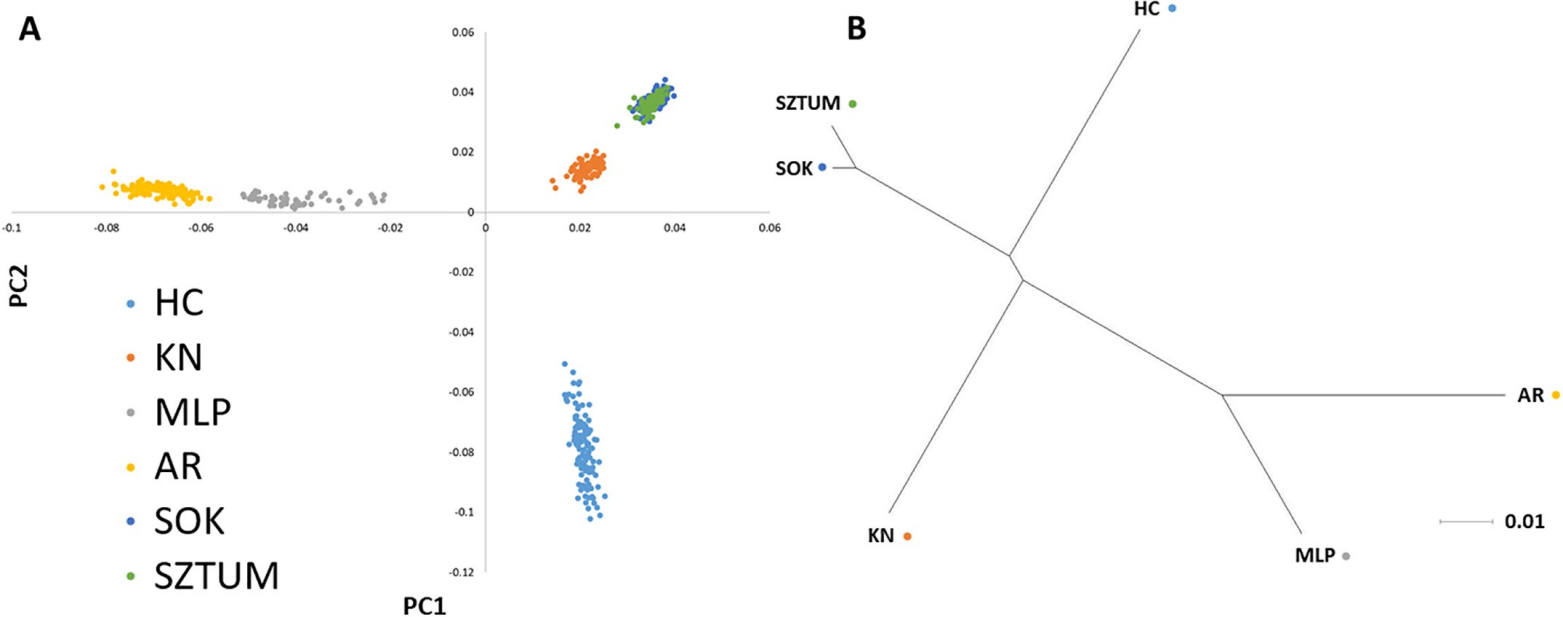
Genetic differentiation of the studied horse breeds visualized using principal components analysis (A) and F_ST_-based cladogram (B). Principal components analysis was performed based on SNP genotypes. The cladogram was created based on weighted F_ST_ distances as presented in Table 2. Neighbor Joining method was used to order the breeds according to their relative genetic distances.

The data filtering allowed obtaining a common set of 52,023 SNPs polymorphic across the whole population with a mean inter-marker distance of 43.0 kb (±45.1). The number of polymorphic SNPs (MAF>0.01) per breed ranged from 47,495 to 50,775 in HC and MLP breeds, respectively. The average MAF across all SNPs was the lowest in KN (0.214) and the highest in MLP (0.262) breed. The averaged observed heterozygosity per breed ranged from 0.296 in KN to 0.353 in MLP (Table 1). Mean and weighted overall pairwise F_ST_ distances were the lowest between both draft horse breeds (0.012 and 0.014) and the highest level of genetic differentiation was found between the Arabians and the primitive or draft horses (Table 2). The breeds differentiation was additionally visualized using the principal component analysis based on SNP genotypes and a cladogram of mean pairwise F_ST_ distances created using the neighbor joining method (NJ) (Fig 1). The PCA analysis showed clear separation of genetic profiles of both light horses from remaining breeds and a clearly distinct genetic profile of Hucul horses, while the NJ method showed the visible similarity of genetic profiles within major horse types with an exception of primitive horses, which were clearly separated (Fig 1).

**Table 1:** SNP panel polymorphism characteristics.

Breed	No of SNPs	Monomorphic	Low frequency variants (0<MAF<0.01)	Polymorphic SNPs (MAF>0.01)	Mean MAF	Observed heterozygosity	Expected heterozygosity	Mean inter-marker distance (kb)
AR	52,023	2,636	1,193	48,194	0.232	0.316	0.311	44.18
HC		3,322	1,206	47,495	0.216	0.302	0.293	
KN		3,252	821	47,950	0.214	0.296	0.292	
MLP		673	575	50,775	0.262	0.353	0.348	
SOK		966	1,495	49,562	0.215	0.296	0.295	
SZTUM		1,435	1,226	49,362	0.214	0.297	0.294	

**Table 2:** Mean (above diagonal) and weighted (below diagonal) pairwise FST distances for the studied horse breeds.

		Mean FST					
	Breed	AR	HC	KP	MLP	SOK	SZTUM
Weighted FST	AR	x	0.121	0.122	0.080	0.122	0.122
	HC	0.148	x	0.093	0.117	0.083	0.084
	KP	0.150	0.112	x	0.113	0.077	0.077
	MLP	0.085	0.128	0.127	x	0.111	0.106
	SOK	0.151	0.083	0.094	0.126	x	0.012
	SZTUM	0.150	0.099	0.093	0.123	0.014	x

### Breed-specific selection signatures

**Fig 2:**
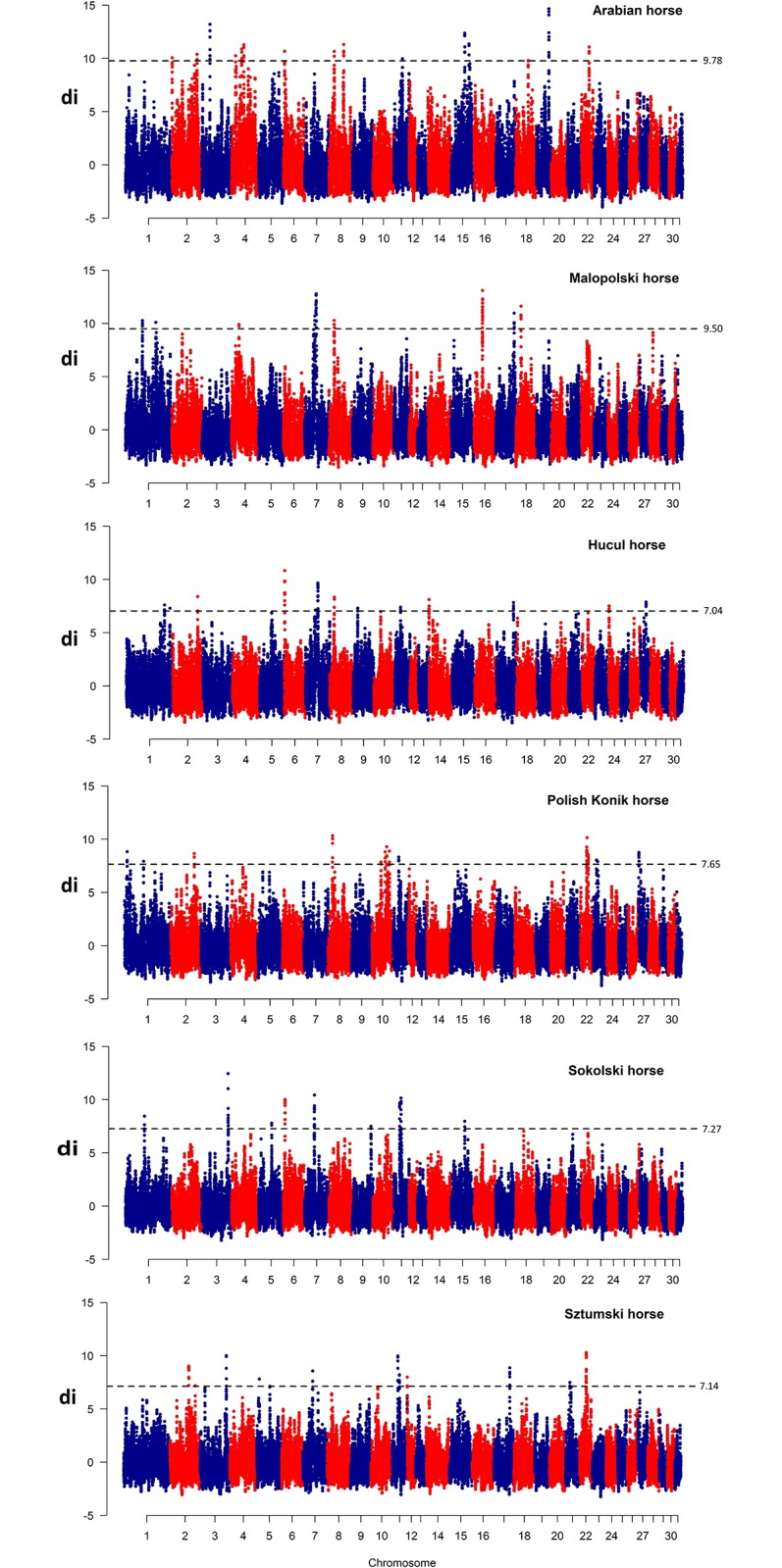
Selection signatures among the studied horse breeds. D_i_ values for comparison between each breed and remaining breeds were plotted against centered genomic positions of the analyzed d_i_ windows. The blue dashed line indicates 99.9% of the highest d_i_ values.

Signatures of diversifying selection among the studied horse breeds were detected based on the breed-normalized pairwise F_ST_ distances (di) (S1 File). After smoothing of the obtained d_i_ values by moving average, top 0.1% of the observations were considered as the most pronounced selection signals associated with breed specific features. The merging of overlapping regions allowed for detection from 10 (MLP, SOK) to 15 (KN) genomic loci with strong selection signals per breed with a size ranging from 163.9 kb to 4.4 Mb (Table 3). The highest number of the strongest selection signals across all breeds was detected on ECA1 and ECA11 and only single regions were detected on ECA12, 14, 16, 19, 21 and 24. Several genomic regions with strong section signals overlapped between different breeds and these regions were located on chromosomes 1, 2, 3, 7, 8, 11, 15 and 22 (Table 3, Fig 2). The most commonly observed selection signal common for different breeds (HC, KN, SOK, SZTUM) was detected on ECA11 between 22.9 and 23.7 Mb of the chromosome sequence.

**Table 3:** Genome regions spanning the top 99.9 percentile of di values representing the strongest diversifying selection signals between the studied horse breeds.

Breed	CHR	start	Stop	Size
AR	2	1,624,948	1,973,143	348,195
	2	101,234,639	101,658,575	423,936
	3	32,433,254	32,870,018	436,764
	4	16,091,738	16,486,662	394,924
	4	40,341,969	40,854,832	512,863
	4	48,962,364	49,486,277	523,913
	6	4,845,026	5,072,913	227,887
	8	21,048,394	21,430,992	382,598
	8	59,193,971	59,722,834	528,863
	11	34,293,691	34,694,290	400,599
	15	54,233,062	54,834,053	600,991
	15	71,304,187	71,920,250	616,063
	18	49,493,092	50,255,137	762,045
	19	50,182,286	51,212,610	1,030,324
HC	1	152,621,838	153,113,818	491,980
	1	174,446,252	174,904,138	457,886
	2	100,516,619	100,788,428	271,809
	6	1,212,183	2,028,427	816,244
	7	40,085,801	40,454,393	368,592
	7	49,901,427	51,046,996	1,145,569
	8	17,883,095	18,489,045	605,950
	9	18,212,591	18,700,974	488,383
	11	23,158,971	23,712,507	553,536
	14	973,820	2,251,732	1,277,912
	17	67,810,418	68,213,301	402,883
	24	2,078,405	2,409,598	331,193
	27	23,060,597	23,534,561	473,964
KN	1	9,825,107	10,332,546	507,439
	1	75,808,309	76,154,827	346,518
	2	93,508,775	93,977,695	468,920
	8	17,883,095	18,611,435	728,340
	10	35,789,240	36,007,871	218,631
	10	52,072,347	52,549,569	477,222
	10	58,667,764	59,059,229	391,465
	10	69,514,973	69,856,620	341,647
	11	23,158,971	23,712,785	553,814
	22	24,711,960	25,203,806	491,846
	22	26,260,281	26,769,595	509,314
	22	30,647,730	31,272,127	624,397
	23	14,554,518	14,978,574	424,056
	23	18,764,766	19,132,094	367,328
	27	1,149,556	1,896,914	747,358
MLP	1	67,781,374	68,262,049	480,675
	1	121,619,834	121,783,765	163,931
	4	29,496,419	30,029,942	533,523
	7	41,537,197	42,121,193	583,996
	7	44,416,041	48,776,083	4,360,042
	8	20,947,045	21,430,992	483,947
	8	21,695,072	22,112,785	417,713
	16	34,084,269	34,987,389	903,120
	17	74,057,493	74,788,851	731,358
	18	20,843,949	21,361,957	518,008
SOK	1	75,808,309	76,229,305	420,996
	3	104,821,176	106,074,186	1,253,010
	5	52,447,911	52,699,832	251,921
	6	6,293,623	6,508,480	214,857
	7	39,645,983	40,575,820	929,837
	9	75,386,842	75,620,809	233,967
	11	22,985,500	23,712,785	727,285
	11	28,738,223	29,312,157	573,934
	11	29,629,969	29,933,173	303,204
	15	54,468,973	54,773,734	304,761
SZTUM	2	75,001,575	75,861,363	859,788
	2	101,372,126	101,658,575	286,449
	3	105,046,433	105,830,625	784,192
	5	9,797,727	10,581,432	783,705
	7	40,036,320	40,454,665	418,345
	11	22,985,500	23,732,591	747,091
	11	29,561,696	30,016,859	455,163
	12	20,064	276,525	256,461
	17	63,828,104	64,275,079	446,975
	21	18,007,980	18,348,602	340,622
	22	25,951,536	26,850,432	898,896

To analyze gene content of the genome regions spanning the strongest detected selection signals (top 0.1% of di values), each region was expanded by 25 kb on up- and downstream directions to account for potentially linked genes. This allowed for detection from 65 (SOK) to 169 (MLP) ENSEMBL genes per breed (S2 File). The analysis of genes common for different breeds showed that as many as 18 genes were common for HC, KP, SOK and SZTUM breeds and no common genes were observed between MLP and both the primitive and draft horses (S3 File) [25]. The functional classification of well-annotated genes using Panther software according to GO terms showed that all the genes detected across breeds (506 unique genes) were mainly involved in cellular processes (32% of genes) like e.g.: cell communication and cell cycle or metabolic processes (22%), including: primary metabolic processes (89 genes), nitrogen compound metabolic processes (50 genes) or phosphate-cogitating compound associated processes (28 genes). All the genes were connected with a large number of Panther pathways, for which the highest number of genes was connected with inflammation mediated by chemokine and cytokine (8 genes), TRH receptor signaling, TGF-beta signaling, CCRK signaling map or GRH receptor signaling pathway (5 genes in each).

When functional classification was performed for individual breeds, visible differences in the enriched GO categories were detected (S4 File). The pathway analysis performed on our dataset for genes found in the Arabian horses showed the presence of some genes connected inter alia with: ATP synthesis coupled electron transport (*COX4I1*), bile secretion *(ABCG8*, *ABCG5*, *ADCY1*), fat digestion, oocyte meiosis (*ADCY1*, *ANAPC7*), ovarian steroidogenesis (*ADCY1*) and insulin signaling (*CBLB*) or secretion (*ADCY1*) pathways. Genes detected in the Malopolski horse were associated with largely similar biological pathways as those found in the Arabian horses and included inter alia: progesterone-mediated oocyte maturation (*PRKACA*, *ANAPC5*, *ANAPC7*), oocyte meiosis (*PRKACA*, *ANAPC5*, *ANAPC7*), fatty acid metabolism (*TECR*), but also covered other processes associated with immune system functions, like e.g.: leukocyte transendothelial migration (*MYL2*), antigen processing and presentation (*PRKAC*A) and inflammatory mediator regulation of TRP channels (*PRKACA*, *PRKCD*). Within selection signatures characteristic for the primitive Hucul breed, we found several genes involved inter alia in processes like: olfactory transduction (*LOC100066263*, *LOC100066541*, *LOC100055475*, *LOC100066487*, *LOC100060476*, *LOC100060509*, *LOC100066238*), cardiac muscle contraction (*ACTC1*) and alanine, aspartate and glutamate metabolism (*GFPT2*, *CPS1*). Among 100 genes detected in the strongest selection signatures found in the Polish Konik horse we detected genes connected e.g. with: inflammatory mediator regulation of TRP channels or T cell receptor signaling pathway (*PLCG1*), chemokine signaling pathway (*GNG4*) and glycerolipid metabolism (*LPIN3*). The genes detected in the draft Sokolski horses were connected inter alia with: cardiac muscle contraction (*ATP1A1*, *CACNB1*), bile secretion (*ATP1A1*, *ABCG8*), fat digestion/absorption (*ABCG8*) and insulin secretion (*ATP1A1*), so with pathways largely similar to those found in the light horses. Genes associated with diversifying selection signatures in the Sztumski horse were connected for example with: prolactin signaling pathway (*CWC25*), cytokine-cytokine receptor interaction (*LASP1*) or general metabolism (*PCGF2*) (S5 File).

### Signatures of diversifying selection between major horse types

To identify genome regions differentially fixed between different major horse types, d_i_ values were calculated between pairs of breeds assigned to three categories: light (AR, MLP), primitive (ponies) horses (HC, KN) and draft horses (SOK, SZTUM). The obtained d_i_ values were then smoothed by sliding window of 10 SNPs and 0.1% of the highest observations was analyzed for gene content (S6 File).

**Fig 3:**
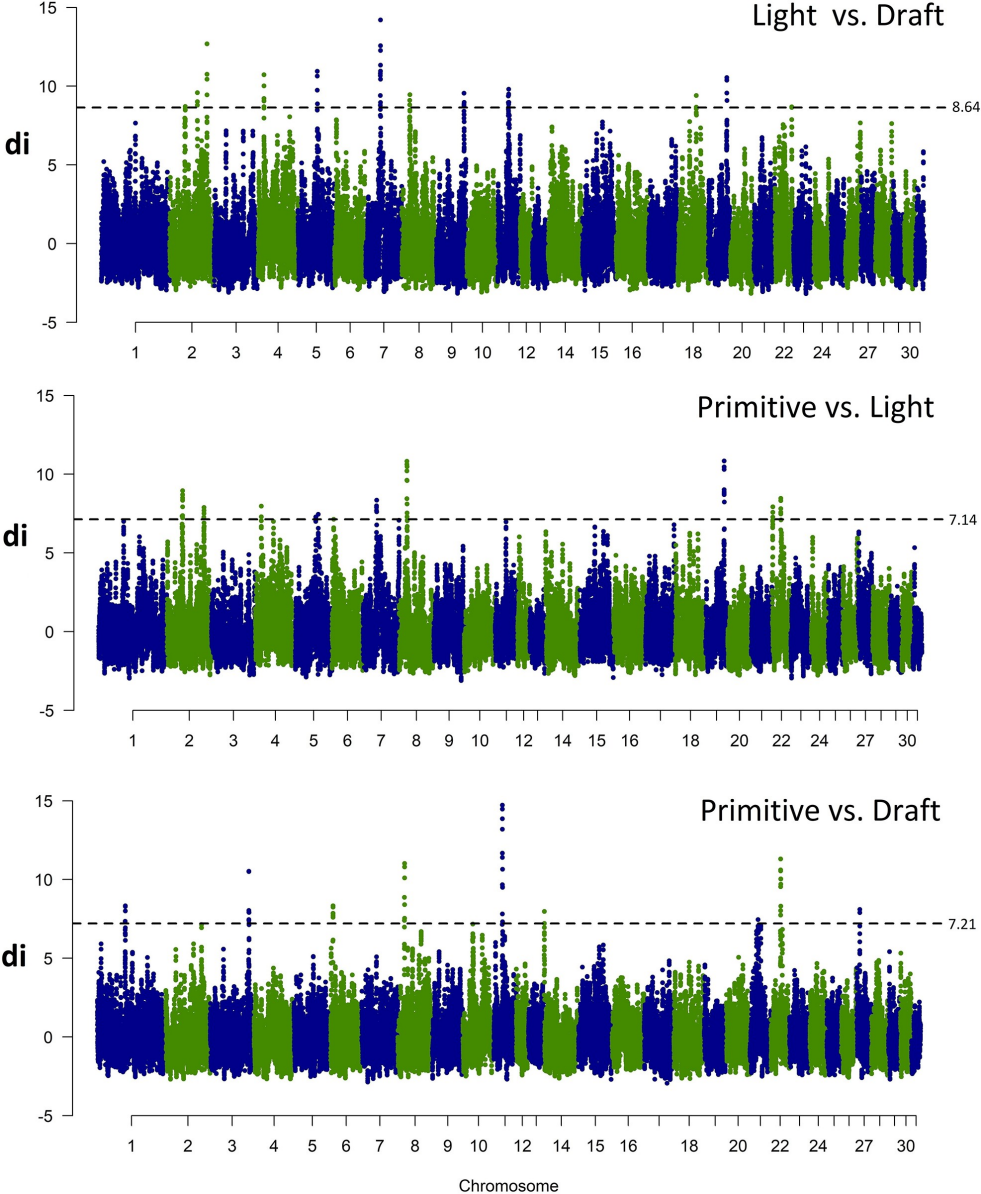
Selection signatures among the major studied horse types. D_i_ values for comparison between major horse types were plotted against centered genomic positions of the analyzed d_i_ windows. The blue dashed line indicates 99.9% of the highest d_i_ values.

This comparison revealed 14 genome regions strongly differing in alleles frequency between the light and draft horses, 11 regions differing between the light and primitive horses and 9 differing between the primitive and draft breeds. The highest number of such divergently selected regions was detected on ECA2 (6 regions), 8 and 22 (4 regions on each). The size of individual regions ranged from 124.2 kb to 1.1 Mb. The regions directly overlapping between at least two different comparisons were found on ECA2, 4, 8, and 19 and these overlaps were found for comparisons between the light horses and two remaining horse types (Table 4). The comparison of selection signature plots (Fig 3) revealed similar pattern of allele frequency differences between the light and two other horse types and distinct pattern of frequency differences between the primitive and draft horses.

**Table 4:** Genome regions spanning the top 99.9 percentile of di values representing the strongest diversifying selection signals between the major studied horse types.

Chr	Start	End	Size
Light vs. Draft			
2	42,283,588	42,693,342	409,754
2	75,104,992	75,633,717	528,725
2	101,239,026	101,659,052	420,026
4	15,870,441	16,546,202	675,761
5	52,350,393	52,725,393	375,000
7	39,875,266	40,815,384	940,118
8	21,048,394	21,430,992	382,598
8	21,877,750	22,178,476	300,726
9	75,379,414	75,680,103	300,689
11	28,762,301	29,038,958	276,657
11	29,561,696	30,111,682	549,986
18	49,493,092	50,504,878	1,011,786
19	50,507,674	51,157,247	649,573
22	45,438,601	45,752,522	313,921
Primitive vs. Light			
2	40,879,307	41,345,280	465,973
2	42,144,042	42,738,837	594,795
2	99,966,856	100,812,765	845,909
4	15,993,651	16,486,662	493,011
5	55,503,606	56,139,444	635,838
5	62,395,899	62,666,145	270,246
7	36,787,932	37,213,908	425,976
8	20,849,134	21,483,539	634,405
19	50,182,286	51,157,247	974,961
22	1,233,709	1,498,126	264,417
22	22,921,048	23,687,775	766,727
Primitive vs. Draft			
1	75,333,244	75,724,769	391,525
1	75,808,309	76,294,080	485,771
3	104,821,176	105,874,361	1,053,185
6	6,384,273	6,508,480	124,207
8	17,879,679	18,615,429	735,750
11	22,807,586	23,892,808	1085,222
14	1,049,008	1,262,151	213,143
21	22,327,128	22,649,580	322,452
22	26,246,949	26,846,925	599,976

Within the detected selection signatures for major horse types we detected in total 220 unique genes–from 77 to 87 for separate comparisons (S7 File). The genes with differentially fixed variants between the light and draft horses were associated with several biological pathways, of which the most pronounced were those connected with progesterone-mediated oocyte maturation, oocyte meiosis (*ANAPC5*, *ANAPC7*, *ADCY1*), cardiac muscle contraction (*MYL2*, *ATP1A1*), adrenergic signaling in cardiomyocytes (*ADCY1*, *MYL2*, *ATP1A1*), salivary or bile secretion, insulin secretion and thyroid hormone synthesis (*ADCY1*, *ATP1A1*). The genes were also connected with biological processes responsible for energy homeostasis, like: ATPase activity or mitochondrial inner membrane function (*ATP5F1E*) (S8 File).

The regions differentially fixed between the primitive and light horses included 77 genes associated with variety of biological pathways among which the most enriched ones were those connected with immune system functions, e.g.: HTLV-I infection (*ETS1*, *ANAPC7*, *ADCY1*, *WNT2B*), ubiquitin mediated proteolysis (*ANAPC7*, *CBLB*, *UBE4B*), bacterial invasion of epithelial cells (*CBLB*, *ARPC3*) and reproduction, like: oocyte maturation and oocyte meiosis (*ANAPC7*, *ADCY1*). The genes were also engaged in biological processes connected with lactate transmembrane transport (*SLC16A1*), pyridine-containing compound metabolic process (*NMNAT1*) and wound healing (*RHOA*) (S8 File).

The genomic regions differing between the primitive and draft horses encompassed 87 different genes among which we detected ones connected with Ras signaling pathway (*GNG4*, *FGF10*), sulfur relay system (*NFS1*), MAPK signaling pathway (*CACNB1*, *FGF10*), cardiac muscle contraction (*CACNB1*) and terpenoid backbone biosynthesis (*GGPS1*). The genes were also annotated to a wide range of biological processes including inter alia: keratinocyte differentiation, angiogenesis and bone development (*MED1*) (S8 File).

Special attention was given to genes located within the strongest signals of diversifying selection between different horse types and involved an analysis of the most divergently selected regions per comparison. For comparison between the draft and light horses we analyzed in details a region on ECA7 (39.8–40.8 Mb), for comparison between the primitive and light horses a region on ECA8 (20.8–21.5 Mb) and for comparison between the draft and primitive horses a region on ECA11 (22.8–23.9 Mb) (Fig 3). Additionally, for these regions we established linkage disequilibrium and haplotype block structure to identify haplotypes potentially being under selection.

Within the region on ECA7 (39.8–40.8 Mb), apart from two pseudogenes and one uncharacterized protein coding gene, we found two genes (namely: *NTM*, coding for Neurotrimin and *OPCML*, coding for opioid binding protein) involved in central nervous system functioning [26]. The analysis of LD and haplotype structure at the analyzed *locus* showed the presence of two haplotype blocks with common haplotypes showing frequency above 0.7 in all analyzed breeds (S9 File). The region also neighbored with a large chromosomal area of high LD in AR and MLP horses located upstream of the analyzed selection signature (40.1–52.5 Mb).

The in-depth analysis of the locus on ECA 8 (20.8–21.5 Mb) revealed the presence of 14 genes within the regions, including one uncharacterized protein. The remaining genes included: *MYL2*, *CCDC63*, *PPP1CC*, *HVCN1*, *TCTN1*, *PPTC7*, *RAD9B*, *VPS29*, *FAM216A*, *GPN3*, *ARPC3*, *ANAPC7*, *ATP2A2*. The analysis of LD at the ECA8 locus showed rather moderate linkage and unambiguous haplotype block structure, however, some overlapping haplotype blocks with frequency higher than 0.5 were found for three out of four compared breeds (S10 File).

**Fig 4:**
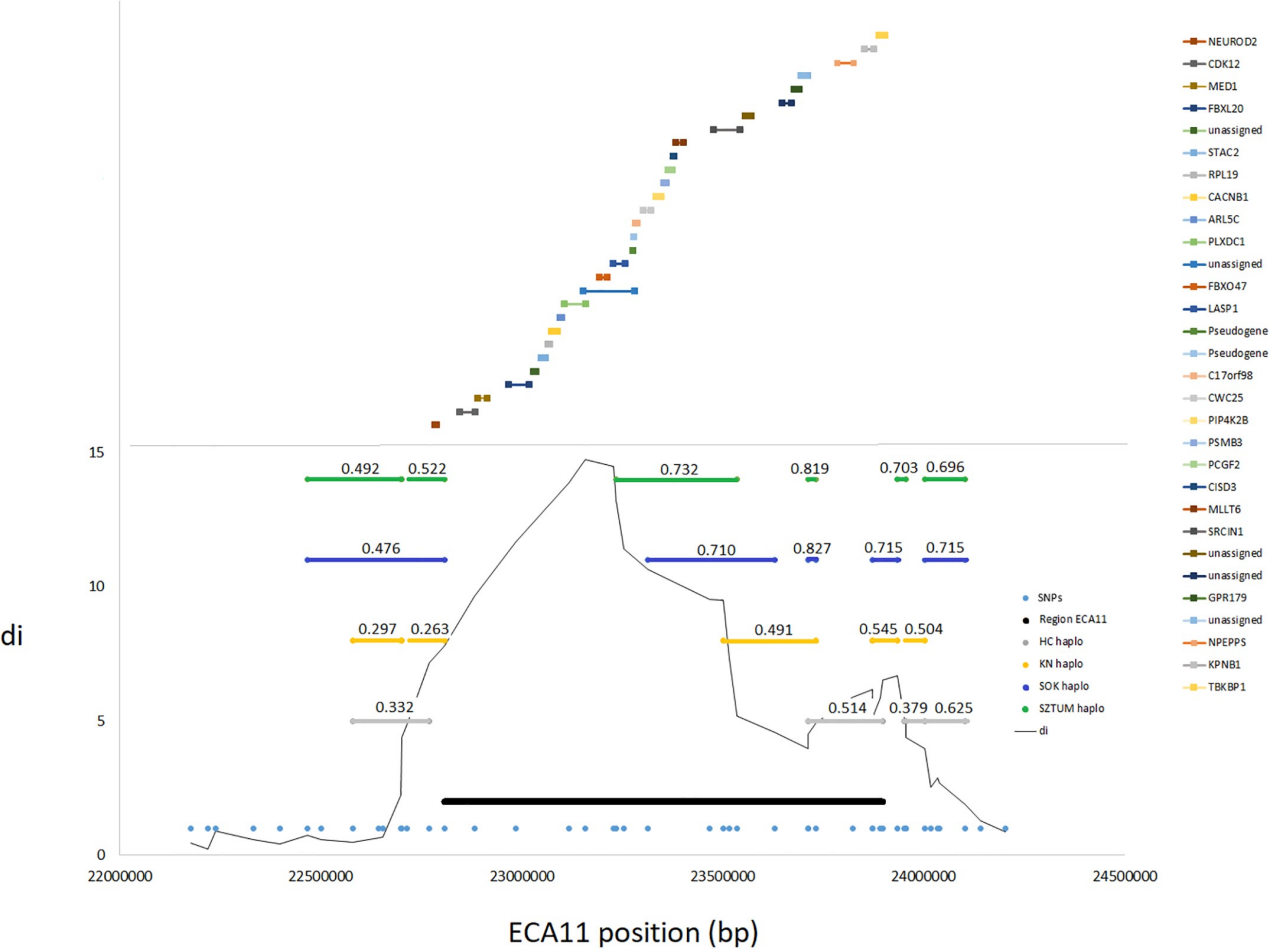
The strongest selection signal at the ECA11 locus spanning the region divergently selected between primitive and draft horses. The graph presents the genomic position of the ECA11 locus, haplotype blocks found in separate breeds along with the frequency of the most common haplotype. The genomic positions of genes annotated directly (±25kb) at the region are also marked.

Within the large region with the highest differences in alleles frequency between the draft and small size primitive horses on ECA11 (22.8–23.9 Mb), we detected 30 different genes (including two pseudogenes and five uncharacterized proteins). One of them, *LASP1* gene, was previously proposed as a candidate gene for size in horses [20]. The analysis of LD structure at the locus showed rather fast linkage disequilibrium decay and haplotype block structure difficult to associate with the selection signal (Fig 4). Nevertheless, overlapping haplotype blocks were detected in the draft horses, spanning haplotypes with frequency exceeding 0.7.

### Diversifying selection signatures at candidate gene loci for size and gene responsible for coat color dilution

**Fig 5:**
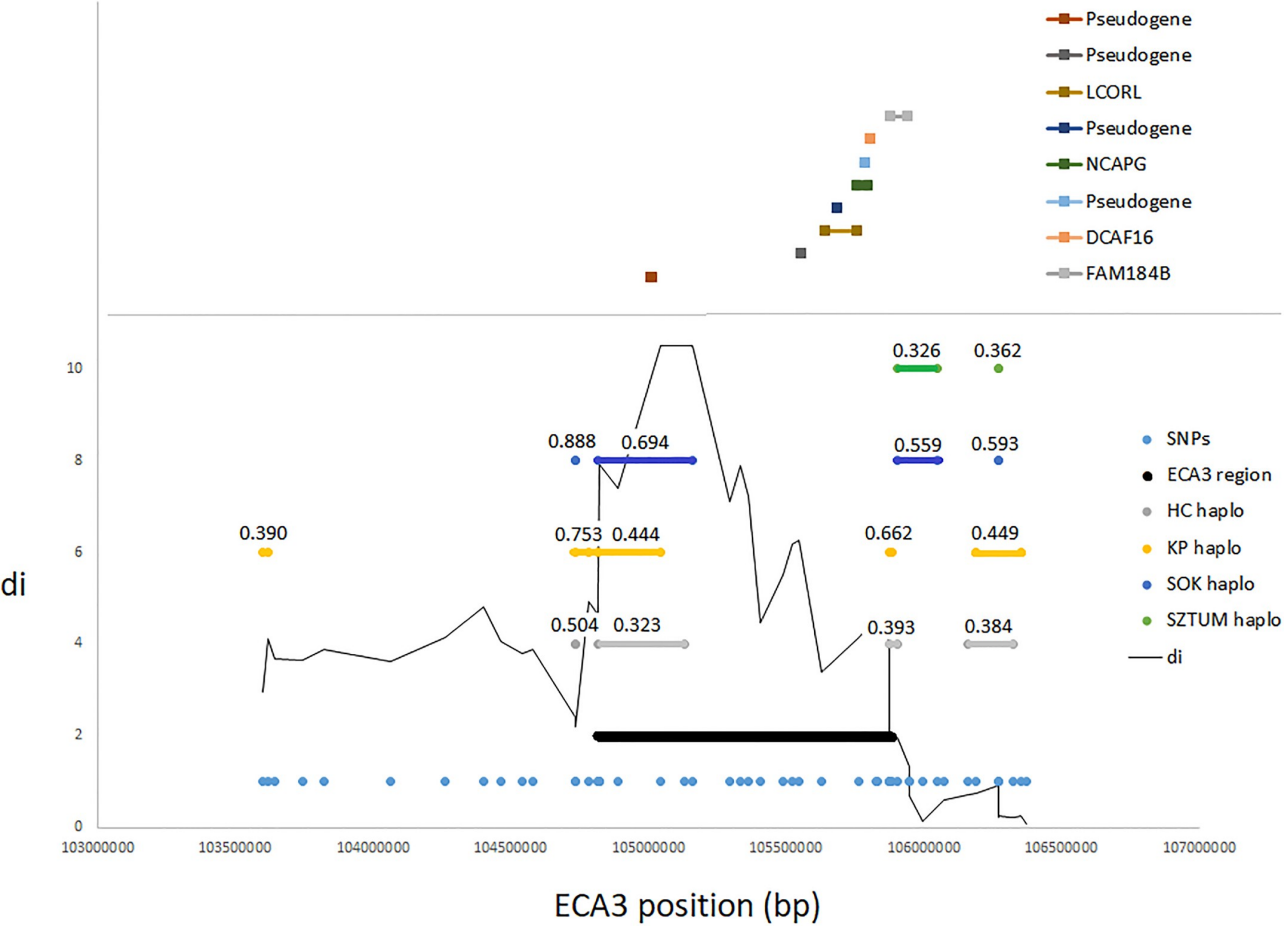
A selection signal on ECA3 associated with the candidate *LCORL/NCAPG* locus for body size in the horse. The graph presents the genomic position of the candidate ECA3 locus, haplotype blocks found in separate breeds along with the frequency of the most common haplotype. The genomic positions of genes annotated directly (±25kb) at the region are also marked.

In previous studies, the ECA3 locus encompassing *LCORL* and *NCAPG* genes was shown to be differentially selected between several draft and miniature horse breeds [2] and was also presented as explaining a significant portion of genetic variance for this trait in an across-breed study [20]. In our results, one of the four strongest diversifying selection signals detected between the small size primitive horses and draft horses, encompassed a previously described locus on ECA3 (104.8–105.9 Mb), spanning both mentioned genes. The analysis of the haplotypes structure at the locus showed overlapping haplotype blocks for three of the analyzed breeds (KP, HC, SOK), however, they spanned a neighboring pseudogene locus rather than *LCORL/NCAPG* positions (Fig 5). This observation may be connected with the possible location of functional variants in the upstream region of *LCORL* (*NCAPG* is transcribed in opposite direction), possibly influencing its promoter activity.

**Fig 6:**
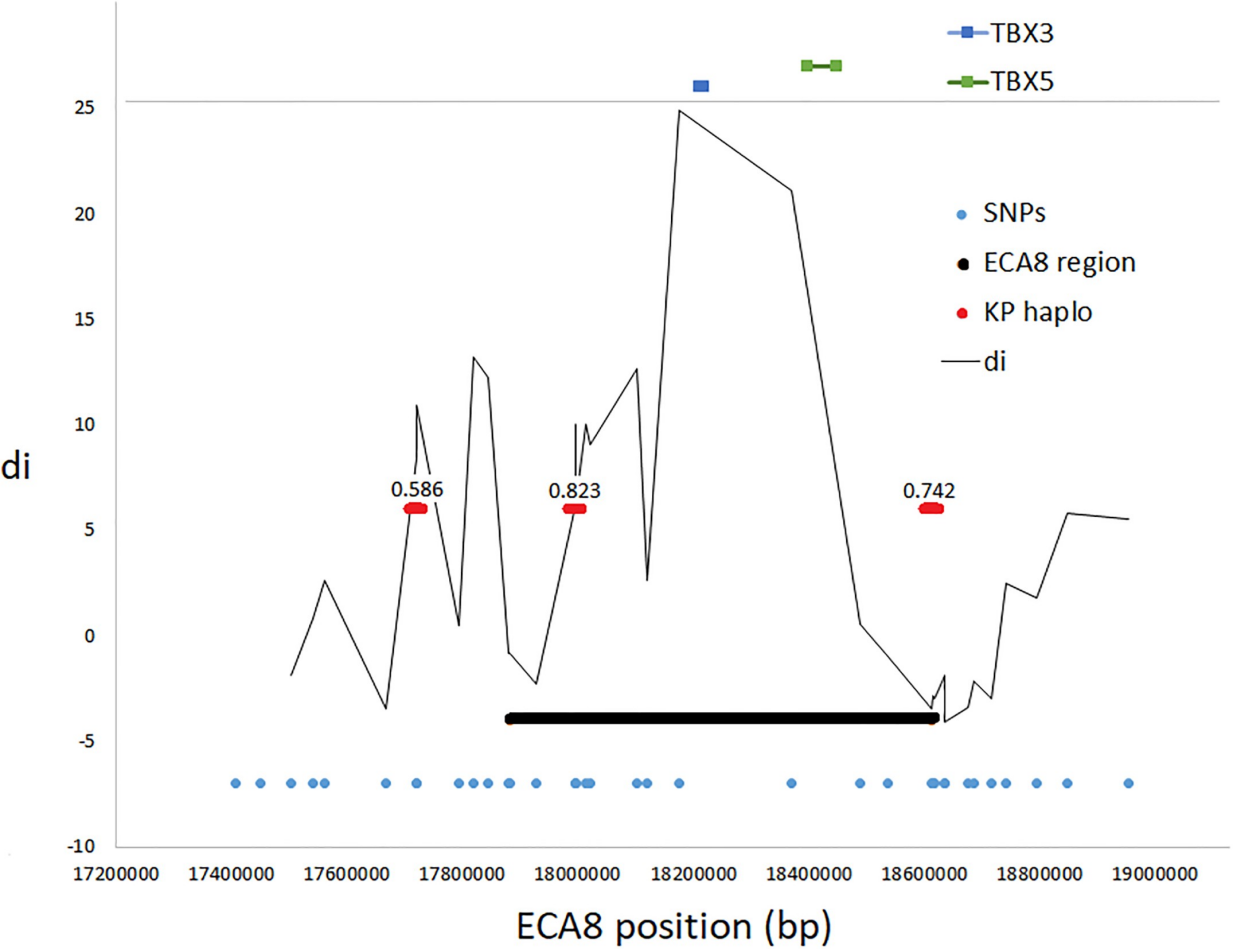
A selection signal on ECA8 associated with the *TBX3* locus responsible for dun coat color dilution in the Polish Konik horse. The graph presents the genomic position of the candidate ECA8 locus and haplotype blocks found in the Polish Konik horse along with the frequency of the most common haplotype. The genomic positions of genes annotated directly (±25kb) at the region are also marked.

Another locus which was analyzed in details was a genome region on ECA8 spanning *TBX3* gene. Previous studies showed that the dun phenotype (which is characteristic for the studied Polish Konik horse) is related to mutations occurring in *TBX3* gene [21,27]. The analysis of diversifying selection signatures characteristic for individual breeds performed in this study showed that the strongest selection signal found in the Polish Konik directly overlapped with this locus (ECA8: 17.9–18.6 Mb). At the locus, we found the low level of LD and poor haplotype structure, however, the detected d_i_ signal almost exactly matched the *TBX3* genomic position (Fig 6).

## Discussion

In this study we performed a genome-wide scan for diversifying selection signatures in horse breeds representing performance, exterior and size selected animals. As a comparative population we included primitive, extensively selected horses with well-developed primitive characteristics like: good fertility, disease resistance and adaptation to harsh environmental conditions [28]. Many genome regions divergently selected between separate breeds were identified in this study as well as selection signatures characteristic for specific horse types (light, draft and primitive). This allowed for an identification of several candidate genes and associated metabolic pathways which may be potentially responsible for the divergent phenotypes of the studied breeds. To allow comprehensive analysis of the obtained results we focused only on the genome regions with the strongest selection signals, presumably being near fixation in separate breeds and spanning variants responsible for the well-established (fixed) breed-specific traits. To manage a variety of genes found within the selection signals we performed a pathways analysis aimed at the identification of enriched processes. Having in mind that the detected selection signatures are associated with a variety of phenotypic features differentiating the studied breeds (which are conditioned by complex molecular mechanisms), we expected only very few genes connected with separate biological processes in the enrichment analysis. Nevertheless, this analysis allowed us to reduce the complexity of the obtained data and allowed us to search for the pathways and underlying genes potentially being the targets of diversifying selection.

The highest number of the strong diversifying selection signals across all breeds was detected in this study on ECA1 and ECA11. These autosomes were previously shown to contain loci affecting size in horses, which both were detected using population genetics [2,16] and quantitative genomics methods [20]. Several genomic regions with strong selection signals overlapped between different breeds and these regions were located on chromosomes 1, 2, 3, 7, 8, 11, 15 and 22 (Table 3, Fig 2). The most commonly observed selection signal common for different breeds was detected on ECA11 between 22.9–23.7 Mb of the chromosome sequence. This region was previously shown as overlapping with the *LASP1* gene locus with a presumed effect on growth and body size traits [2,20].

The analysis of linkage disequilibrium at the most pronounced selection signals found in this study on ECA7, 8 and 11 between major horse types showed rather low level of LD and a poorly conserved haplotype structure in the analyzed breeds, suggesting that the variants selected at the regions are evolutionarily old and their frequency increased during breeds formation rather than under the influence of recent selection. This can be supported by the fact that an high LD expected in regions bearing variants under strong ongoing positive selection [29], actually decayed at the analyzed loci as a result of repeated meiosis and recombinational mechanisms acting over several generations. This is in accordance with the suggestions that different statistical methods, like e.g. extended haplotype homozygosity tests are more suitable to detect selection signatures associated with ongoing selection directed at new functional variants [30]. Nevertheless, selection signals associated with variants which are not fully fixed in the studied populations should be present among signatures that did not reach the applied in this study stringent threshold of 99.9% of the highest d_i_ values and more in-depth analysis of our data would allow detecting variants currently segregating in the studied populations [31].

### Diversifying selection signatures among major horse types

Within the region on ECA7 (39.8–40.8 Mb), divergently selected between the draft and light horses, apart from two pseudogenes and one uncharacterized protein coding gene we found two genes (namely: *NTM*, coding for neurotrimin and *OPCML*, coding for opioid binding protein) that were involved in central nervous system functioning. The study performed in humans suggested that *NTM* gene (coding for Neurotrimin) locus is associated with the level of IQ and two other genome wide association studies (GWAS) reported an association between *NTM* variation and cognitive function performances in humans [32, 33]. The diversifying selection signature at this locus between the draft and light horses may potentially contribute to their differing temperaments and/or potentially, the ability to develop different gaits as a function of motor coordination managed by the brain.

The locus on ECA 8 (20.8–21.5 Mb) with clear divergences in alleles frequency between the primitive and light horses encompassed 14 genes, including one uncharacterized protein. Among the genes as potential selection candidate we suggest *MYL2* (Myosin Light Chain 2) gene (detected at the peak of the selection signature), which is coding for a contractile protein that plays a significant role in heart development and contraction [34, 35]. The mutation in *MYL2* was shown to be responsible for hypertrophic cardiomyopathy (HCM) in humans [36]. The association of the gene with heart contraction and development suggests its potential role in physical effort and advocates selection pressure on its locus in light horses in terms of their performance and endurance which are strongly dependent on heart and lung efficiency [37]. The *MYL2* gene was also identified in a genome-wide study on quantitative trait loci affecting show-jumping performance in Hanoverian warmblood horses [38].

Interestingly, one of the genome regions on ECA5 differentiating light and primitive horses encompassed *SLC16A1* (Solute Carrier Family 16 Member 1) gene. The MCT1 protein encoded by this gene catalyzes the movement of different monocarboxylates such as lactate and pyruvate across the plasma membrane [39]. In a previous study, Ropka-Molik et al. [40] showed that *SLC16A1* gene expression is stimulated during training regimens preparing horses for flat racing. In the studied Arabian horses they detected that the *SLC16A1* expression gradually increased in muscle tissue, starting from resting conditions up to top training form [40]. Furthermore, the performed association analysis showed that there was a significant association between g.55589063T>G SNP located in the 5’UTR of *SLC16A1* gene and the selected racing results [41]. All these evidences suggest the potential significance of *SLC16A1* gene and MCT1 protein for horse performance and that the detected selection signal associated with genetic differences between primitive and light horses may be a result of selection (applied especially in the Arabian and Malopolski horses) towards the improvement of racing abilities.

Within the large region on ECA11 (22.8–23.9 Mb) divergently selected between the primitive (small size) and draft horses (so presumably responsible for size), we detected 30 different genes. Among the genes we detected *LASP1* gene, previously suggested as a candidate gene for size in horses [20]. However, some doubts were also associated with this gene as a candidate for size [16] probably due to the insufficient knowledge on this gene functions, hampering clear description of its role in body size regulation [42].

Apart from selection signatures representing differentiation of horse types, a detailed analysis was performed to detect genome regions divergently selected in individual horse breeds. The analysis was aimed at the detection of candidate genes affecting breed-specific characteristics.

### Selection signatures found in light horses (Arabian and Malopolski)

Both the Arabian and Malopolski horses are characterized by several common features and are lightweight horses with a breeding purpose mainly directed at aesthetics, racing performance and gait quality. Our study allowed identifying in these horses selection signatures spanning genes involved in regulation of pathways associated with metabolic processes and energy production from lipids (such as: bile secretion, fat digestion and absorption, fatty acid metabolism and regulation of lipolysis in adipocyte) or from carbohydrates (insulin secretion, insulin signaling pathway) (S2 and S5 Files). Several previous studies based on animal models and humans suggest that exercise training improves lipid and cholesterol metabolism [43–45]. According to Meissner et al. [46], physical activity increase bile acid synthesis and as a result, increase fatty acid absorption in exercise-trained animals. Furthermore, it was also shown that exercise increases the insulin-signaling cascade activity, which can be associated with alterations in insulin sensitivity [47]. Similarly, Ropka-Molik et al. [48] pointed out the significant role of mechanisms modulating glucose uptake and lipid metabolism in maintaining body homeostasis during long-term exercise in horses, thus providing strong grounds that elements of this mechanism presumably are targets of selection in light horses. Insulin receptor signaling pathway was also shown to be enriched by genes detected in a selection sweeps found in a recent study employing next generation sequencing data from 52 horses of different breeds [49]. This suggests that insulin signaling is a biochemical cascade important for general horse functional features and their element are under selection in various types and horse breeds.

Moreover, our results obtained for the Arabian horses which dominate in endurance riding and racing [50] pinpointed genes classified as involved in vascular smooth muscle contraction *(ADCY1*), taurine and hypotaurine metabolism (*GAD1*) as well as in oxidative phosphorylation (*COX4I1*), that is processes with clear implications for racing and athletic performance. It is well known that blood flow through the muscle (conditioned inter alia by vascular smooth muscle contraction) can change over time and highly increase during maximal exercise [51–53]. This blood flow adjustment is primarily related to increased oxygen demands of the muscle tissue and it is essential for physical performance during racing. The importance of the role of taurine in exercise endurance has been highlighted by many reports. In mice, the individuals that lacked the taurine transporter (TauT) gene and contained severely reduced muscle taurine content exhibited decreased ability to perform physical exercise in both treadmill and forced swimming tests [54, 55]. Furthermore, Dawson et al. [56] and Miyazaki et al. [57] proved that taurine supplementation prolongs the time to exhaustion during treadmill running, by the release of intramuscular taurine into the blood. Processes of oxidative phosphorylation are even more important for physical endurance. The ATP demand rapidly increases to meet the high rate of ATP consumption during rest-to-work transition and hence efficient processes of ATP synthesis are crucial for muscle kinetics [58]. These pieces of evidence suggest that the detected genes connected with vascular smooth muscle contraction, taurine and hypotaurine metabolism as well as oxidative phosphorylation play a significant role in horse athletic performance and are promising candidates for the endurance traits in the studied Arabian horses.

### Selection signatures found in primitive horses (Hucul and Polish Konik)

Hucul and Polish Konik, apart from clear genetic differences (also demonstrated in this study) share several common features resulting from their primitive character and common phylogenic origin. Both these horse breeds are presumed to originate from Eastern European wild horse (Tarpan) and are characterized by several primary features adapting them to living in natural conditions. These features are mainly shaped by natural selection and represent adaptation to survival in harsh environment and are connected with ability for searching food, defense against predators, disease resistance and tolerance of adverse climatic conditions [28].

In Hucul horses, selection signatures overlapped with several genes classified as engaged in olfactory transduction pathway (*LOC100066263*, *LOC100066541*, *LOC100055475*, *LOC100066487*, *LOC100060476*, *LOC100060509*, *LOC100066238*). It is generally thought that the olfactory system is extremely important for the survival of most mammals and is essential for finding foods, avoiding dangers or identifying mates and offspring [59–63]. It helps to survive in harsh environmental/living conditions to which Hucul horses are well adapted.

Our analysis of selection signatures in the Polish Konik breed allowed the detection of a strong selection signal directly at *TBX3* gene locus. This gene was previously reported as being causative for the dun phenotype [21] by affecting expression of *KITLG* gene. There is also strong evidence demonstrating that two single nucleotide polymorphisms within this gene are responsible for coat color dilution and occurrence of three alleles: dun (D), non-dun1 (d1), and non-dun2 (d2) was presented [21]. The detection of the strong selection signal unambiguously associated with this locus in the Polish Konik horse (sole breed with this coat color phenotype among the studied breeds) confirms the involvement of *TBX3* gene in coat color dilution in this breed and the usefulness of the applied population-based approach for detection of functional variants loci in the horse genome.

### Selection signatures detected in draft horses

Cold-blooded horses thanks to their muscling, size, strength and stamina are perfect for field work and transport. In the analyzed heavy draft horses, we detected selection signatures encompassing genes involved in pathways essential for maintaining body homeostasis like: aldosterone-regulated sodium reabsorption pathway responsible for sodium homeostasis and blood volume and blood pressure control [64], as well as mineral absorption or endocrine and other factor-regulated calcium reabsorption pathways, playing a central role in the homeostasis of ions [65]. We also identified genes involved in aforementioned pathways associated with metabolic processes and energy production (such as: bile secretion, fat digestion and absorption, protein digestion and absorption, insulin secretion or metabolic pathways) (S2 and S5 Files). All these mechanisms play a crucial role in regulation of biological and cellular functions that are necessary for maintaining body homeostasis during heavy physical effort, which is one of the most important traits selected in draft horses.

In both studied draft horse breeds, we detected a strong selection signal on ECA3, overlapping with previously described *LCORL/NCAPG* locus affecting size in the horse [2, 20, 66]. These genes were associated with size and growth traits in humans and livestock animals. It was shown that the *DCAF16-NCAPG* region is a QTL for the average daily gain trait in cattle [67]. Furthermore, the region including *NCAPG* and *LCORL* loci was reported to be involved in the determination of size, including both height and mass in cattle [68–72] as well as in pigs [10]. The recent studies confirmed also a significant link between these loci and body height in several horse breeds, including Belgian draft horses [2,20,73–76].

## Conclusions

Summarizing, in this study we applied a population differentiation-based approach to detect genomic regions divergently selected between six breeds representing light, draft and primitive horses. The analysis of the most pronounced selection signals allowed us to detect several genes connected with processes potentially important for breeds phenotypic differentiation and associated with energy homeostasis during physical effort, heart functioning, neuron development, fertility, disease resistance and motor coordination. All these processes are potentially important for the traits selected in the analyzed breeds, especially for athletic performance, health and gait quality. Our results also confirmed the previously described association of loci on ECA3 and ECA11 with the regulation of body size in our draft and primitive (small size) horses. The efficiency of the applied statistical approach was also confirmed by the identification of a strong selection signal in the blue dun Polish Konik horse at the locus of *TBX3* gene, which was previously shown to be causative for dun coat color dilution.

## Supporting information

S1 File D_i_ values obtained for individual breeds. The corresponding centered genomic position of separate d_i_ windows is given.
(XLSX)

S2 File Genes found within regions within the top 99.9% of the highest d_i_ values for individual breeds. (XLSX)

S3 File Venn diagram for genes identified in the most diversified genomic regions between the studied horse breeds (top 99.9% of di values). (PNG)

S4 File Top 10 GO biological processes associated with genes found within the strongest diversifying selection signals for individual breeds. (DOCX)

S5 File KEEG pathways associated with genes found within the strongest signals of diversifying selection for individual breeds. (XLSX)

S6 File D_i_ values obtained for comparison between major horse types. The corresponding centered genomic position of separate d_i_ windows is given.
(XLSX)

S7 File Genes found within regions within the top 99.9% of the highest d_i_ values for major horse types. (XLSX)

S8 File Top 10 KEGG pathways associated with genes found within the strongest diversifying selection signals between major horse types. (DOCX)

S9 File The strongest selection signal at the ECA7 locus spanning the region divergently selected between draft and light horses. The graph presents the genomic position of the ECA7 locus, haplotype blocks found in separate breeds along with the frequency of the most common haplotype. The genomic positions of genes annotated directly (±25kb) at the region are also marked.
(PNG)

S10 File The strongest selection signal at the ECA8 locus spanning the region divergently selected between primitive and light horses. The graph presents the genomic position of the ECA8 locus, haplotype blocks found in separate breeds along with the frequency of the most common haplotype. The genomic positions of genes annotated directly (±25kb) at the region are also marked.
(PNG)

## Abbreviations

AR: Arabian horse
ECA: Horse chromosome (E. caballus)
HC: Hucul horse
KN: Polish Konik horse
LD: Linkage disequilibrium
MAF: Minor allele frequency
MLP: Malopolski horse
NJ: Neighbor joining method
PCA: Principal components analysis
SNP: Single nucleotide polymorphism
SOK: Sokolski horse
SZTUM: Sztumski horse
