# Supplementary figures and images for: A genome-wide scan for diversifying selection signatures in selected horse breeds
Source: PLoS One. 2019 Jan 30;14(1):e0210751. doi: 10.1371/journal.pone.0210751 (PMC6353161; doi:10.1371/journal.pone.0210751)

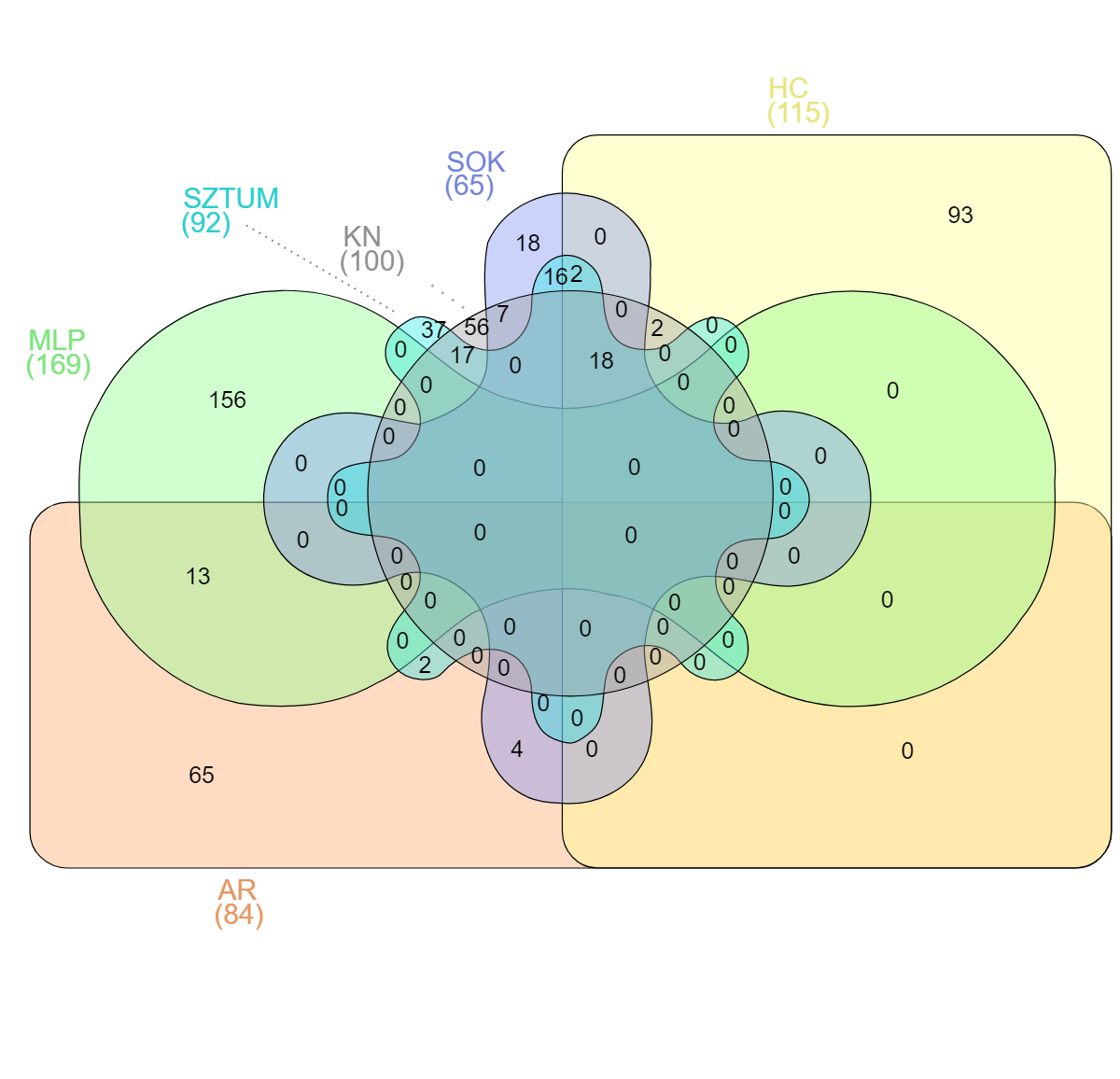

Supplement: S3 File — (PNG) [file pone.0210751.s003.png]

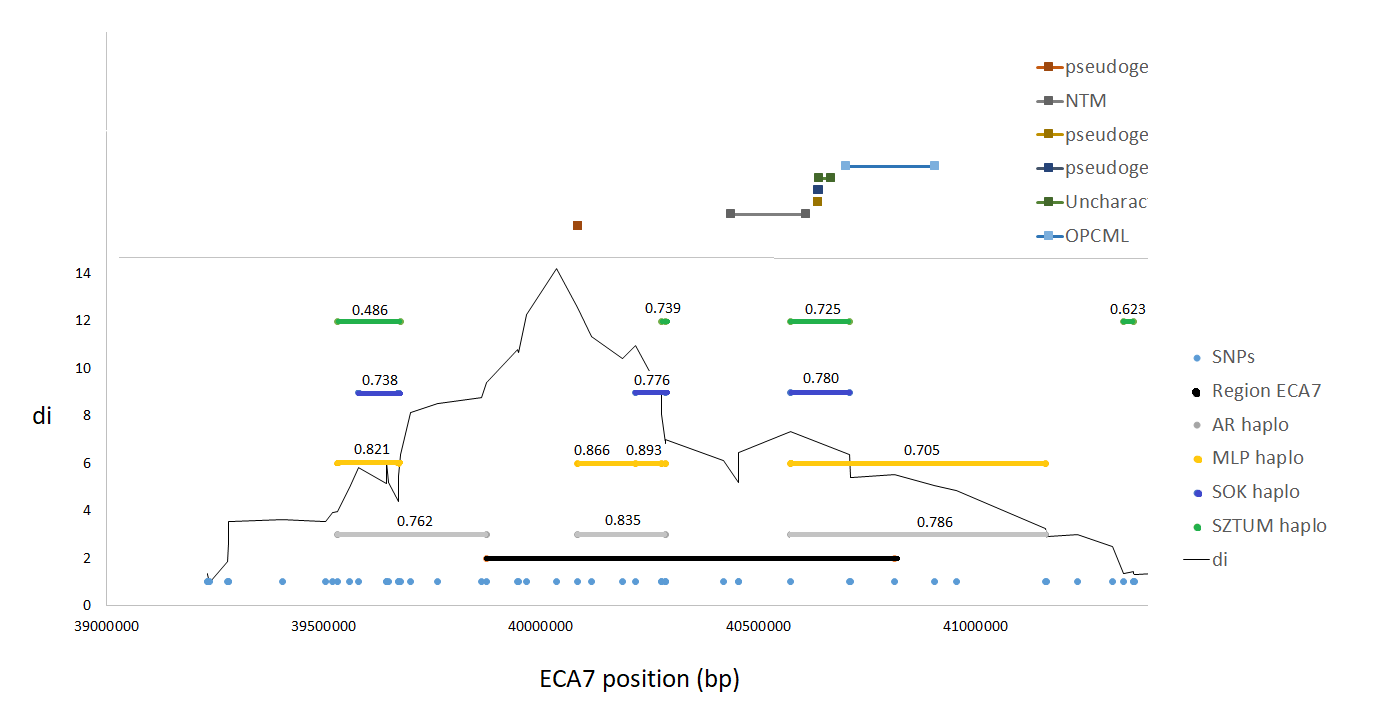

Supplement: S9 File — The graph presents the genomic position of the ECA7 locus, haplotype blocks found in separate breeds along with the frequency of the most common haplotype. The genomic positions of genes annotated directly (±25kb) at the region are also marked. (PNG) [file pone.0210751.s009.png]

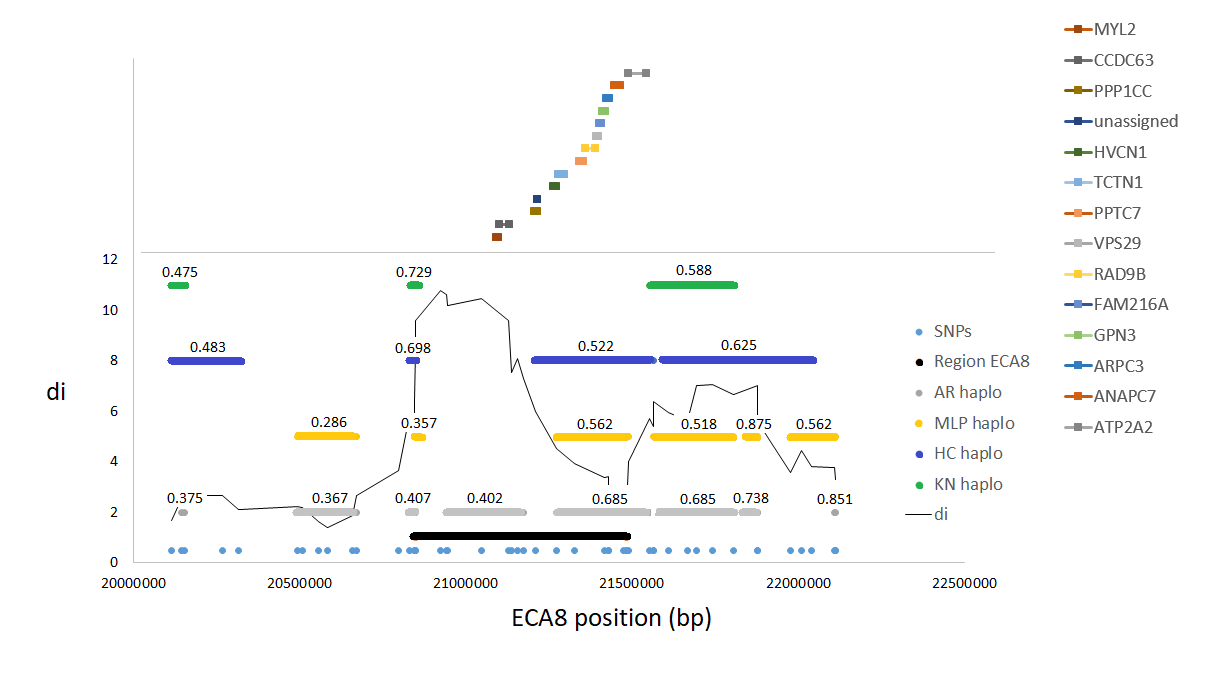

Supplement: S10 File — The graph presents the genomic position of the ECA8 locus, haplotype blocks found in separate breeds along with the frequency of the most common haplotype. The genomic positions of genes annotated directly (±25kb) at the region are also marked. (PNG) [file pone.0210751.s010.png]
