# Supplementary material for: A genome-wide scan for diversifying selection signatures in selected horse breeds
Source: PLoS One. 2019 Jan 30;14(1):e0210751. doi: 10.1371/journal.pone.0210751 (PMC6353161; doi:10.1371/journal.pone.0210751)
Supplement: S4 File — (DOCX) [file pone.0210751.s004.docx]

Top 10 GO biological processes associated with genes found within the strongest diversifying selection signals for individual breeds

| #Term | ID | Input number | Background number | P-Value | Corrected P-Value |
| --- | --- | --- | --- | --- | --- |
| AR | | | | | |
| Cytoskeleton | GO:0005856 | 2 | 62 | 0.016 | 0.210 |
| Carbon-nitrogen ligase activity, with glutamine as amido-N-donor | GO:0016884 | 1 | 5 | 0.018 | 0.210 |
| Cytochrome complex | GO:0070069 | 1 | 5 | 0.018 | 0.210 |
| Negative regulation of organelle organization | GO:0010639 | 1 | 5 | 0.018 | 0.210 |
| Aspartate family amino acid metabolic process | GO:0009066 | 1 | 6 | 0.021 | 0.210 |
| Cytoplasmic part | GO:0044444 | 5 | 521 | 0.022 | 0.210 |
| Nucleus organization | GO:0006997 | 1 | 7 | 0.024 | 0.210 |
| Oxidative phosphorylation | GO:0006119 | 1 | 7 | 0.024 | 0.210 |
| ATP synthesis coupled electron transport | GO:0042773 | 1 | 7 | 0.024 | 0.210 |
| Mitochondrial ATP synthesis coupled electron transport | GO:0042775 | 1 | 7 | 0.024 | 0.210 |
| HC | | | | | |
| Detection of chemical stimulus involved in sensory perception of smell | GO:0050911 | 3 | 10 | 1E-05 | 3E-03 |
| Sensory perception of smell | GO:0007608 | 4 | 69 | 1E-04 | 1E-02 |
| Sensory perception of chemical stimulus | GO:0007606 | 4 | 127 | 9E-04 | 6E-02 |
| Sensory perception | GO:0007600 | 4 | 134 | 1E-03 | 6E-02 |
| Neurological system process | GO:0050877 | 4 | 139 | 1E-03 | 6E-02 |
| Detection of chemical stimulus involved in sensory perception | GO:0050907 | 3 | 67 | 2E-03 | 6E-02 |
| Detection of stimulus involved in sensory perception | GO:0050906 | 3 | 67 | 2E-03 | 6E-02 |
| Detection of chemical stimulus | GO:0009593 | 3 | 71 | 2E-03 | 6E-02 |
| Detection of stimulus | GO:0051606 | 3 | 74 | 2E-03 | 6E-02 |
| System process | GO:0003008 | 4 | 170 | 3E-03 | 7E-02 |
| KN | | | | | |
| Nuclear replication fork | GO:0043596 | 1 | 5 | 0.024 | 0.805 |
| Nucleobase metabolic process | GO:0009112 | 1 | 5 | 0.024 | 0.805 |
| Chromatin remodeling | GO:0006338 | 1 | 6 | 0.028 | 0.805 |
| Hydrolase activity, acting on carbon-nitrogen (but not peptide) bonds | GO:0016810 | 1 | 7 | 0.032 | 0.805 |
| Replication fork | GO:0005657 | 1 | 10 | 0.044 | 0.805 |
| DNA replication | GO:0006260 | 1 | 16 | 0.068 | 0.805 |
| Zinc ion binding | GO:0008270 | 1 | 16 | 0.068 | 0.805 |
| Chromosome segregation | GO:0007059 | 1 | 18 | 0.075 | 0.805 |
| Transition metal ion binding | GO:0046914 | 1 | 21 | 0.087 | 0.805 |
| DNA conformation change | GO:0071103 | 1 | 28 | 0.113 | 0.805 |
| MLP | | | | | |
| Regulation of cell differentiation | GO:0045595 | 2 | 45 | 0.023 | 0.408 |
| Neuropilin binding | GO:0038191 | 1 | 6 | 0.035 | 0.408 |
| Negative chemotaxis | GO:0050919 | 1 | 7 | 0.039 | 0.408 |
| Negative regulation of chemotaxis | GO:0050922 | 1 | 8 | 0.044 | 0.408 |
| Negative regulation of developmental growth | GO:0048640 | 1 | 8 | 0.044 | 0.408 |
| Neural crest cell development | GO:0014032 | 1 | 8 | 0.044 | 0.408 |
| Chemorepellent activity | GO:0045499 | 1 | 8 | 0.044 | 0.408 |
| Neural crest cell migration | GO:0001755 | 1 | 8 | 0.044 | 0.408 |
| Negative regulation of axon guidance | GO:1902668 | 1 | 8 | 0.044 | 0.408 |
| Negative regulation of axon extension involved in axon guidance | GO:0048843 | 1 | 8 | 0.044 | 0.408 |
| SOK | | | | | |
| Cation channel complex | GO:0034703 | 1 | 5 | 0.018 | 0.294 |
| Regulation of calcium ion transport | GO:0051924 | 1 | 5 | 0.018 | 0.294 |
| Regulation of ion transmembrane transporter activity | GO:0032412 | 1 | 6 | 0.021 | 0.294 |
| Regulation of transporter activity | GO:0032409 | 1 | 6 | 0.021 | 0.294 |
| Regulation of transmembrane transporter activity | GO:0022898 | 1 | 6 | 0.021 | 0.294 |
| Regulation of cation transmembrane transport | GO:1904062 | 1 | 6 | 0.021 | 0.294 |
| Regulation of transmembrane transport | GO:0034762 | 1 | 8 | 0.027 | 0.294 |
| Regulation of ion transmembrane transport | GO:0034765 | 1 | 8 | 0.027 | 0.294 |
| Calcium ion transmembrane transport | GO:0070588 | 1 | 8 | 0.027 | 0.294 |
| Regulation of metal ion transport | GO:0010959 | 1 | 8 | 0.027 | 0.294 |
| SZTUM | | | | | |
| Cellular response to topologically incorrect protein | GO:0035967 | 1 | 5 | 0.023 | 0.331 |
| Cation channel complex | GO:0034703 | 1 | 5 | 0.023 | 0.331 |
| Regulation of calcium ion transport | GO:0051924 | 1 | 5 | 0.023 | 0.331 |
| Response to unfolded protein | GO:0006986 | 1 | 5 | 0.023 | 0.331 |
| N-glycan processing | GO:0006491 | 1 | 5 | 0.023 | 0.331 |
| Endoplasmic reticulum unfolded protein response | GO:0030968 | 1 | 5 | 0.023 | 0.331 |
| Cellular response to unfolded protein | GO:0034620 | 1 | 5 | 0.023 | 0.331 |
| Regulation of transmembrane transporter activity | GO:0022898 | 1 | 6 | 0.027 | 0.331 |
| Regulation of ion transmembrane transporter activity | GO:0032412 | 1 | 6 | 0.027 | 0.331 |
| Regulation of transporter activity | GO:0032409 | 1 | 6 | 0.027 | 0.331 |
