# Supplementary material for: A genome-wide scan for diversifying selection signatures in selected horse breeds
Source: PLoS One. 2019 Jan 30;14(1):e0210751. doi: 10.1371/journal.pone.0210751 (PMC6353161; doi:10.1371/journal.pone.0210751)
Supplement: S8 File — (DOCX) [file pone.0210751.s008.docx]

Top 10 KEGG pathways associated with genes found within the strongest diversifying selection signals between major horse types

| #Term | Database | ID | Input number | Background number | P-Value | Corrected P-Value |
| --- | --- | --- | --- | --- | --- | --- |
| Light vs Draft | | | | | | |
| Progesterone-mediated oocyte maturation | KEGG PATHWAY | ecb04914 | 3 | 73 | 0.001 | 0.178 |
| Oocyte meiosis |  | ecb04114 | 3 | 93 | 0.003 | 0.178 |
| Ubiquitin mediated proteolysis |  | ecb04120 | 3 | 111 | 0.005 | 0.178 |
| Adrenergic signaling in cardiomyocytes |  | ecb04261 | 3 | 114 | 0.005 | 0.178 |
| Cardiac muscle contraction |  | ecb04260 | 2 | 50 | 0.011 | 0.203 |
| Thyroid hormone synthesis |  | ecb04918 | 2 | 52 | 0.011 | 0.203 |
| Salivary secretion |  | ecb04970 | 2 | 56 | 0.013 | 0.203 |
| Bacterial invasion of epithelial cells |  | ecb05100 | 2 | 57 | 0.013 | 0.203 |
| Bile secretion |  | ecb04976 | 2 | 59 | 0.014 | 0.203 |
| Gastric acid secretion |  | ecb04971 | 2 | 60 | 0.015 | 0.203 |
| Primitive vs. Light | | | | | | |
| HTLV-I infection | KEGG PATHWAY | ecb05166 | 4 | 206 | 0.006 | 0.425 |
| Ubiquitin mediated proteolysis |  | ecb04120 | 3 | 111 | 0.007 | 0.4245 |
| Bacterial invasion of epithelial cells |  | ecb05100 | 2 | 57 | 0.017 | 0.425 |
| Fc gamma R-mediated phagocytosis |  | ecb04666 | 2 | 61 | 0.019 | 0.425 |
| Dilated cardiomyopathy |  | ecb05414 | 2 | 69 | 0.024 | 0.425 |
| Progesterone-mediated oocyte maturation |  | ecb04914 | 2 | 73 | 0.027 | 0.425 |
| Pancreatic secretion |  | ecb04972 | 2 | 77 | 0.030 | 0.425 |
| Melanogenesis |  | ecb04916 | 2 | 78 | 0.030 | 0.425 |
| Endocytosis |  | ecb04144 | 3 | 217 | 0.039 | 0.479 |
| Oocyte meiosis |  | ecb04114 | 2 | 93 | 0.042 | 0.481 |
| Primitive vs. Draft | | | | | | |
| Sulfur relay system | KEGG PATHWAY | ecb04122 | 1 | 7 | 0.029 | 0.324 |
| Ribosome |  | ecb03010 | 2 | 108 | 0.060 | 0.367 |
| Terpenoid backbone biosynthesis |  | ecb00900 | 1 | 19 | 0.070 | 0.367 |
| Regulation of actin cytoskeleton |  | ecb04810 | 2 | 156 | 0.112 | 0.416 |
| Proteasome |  | ecb03050 | 1 | 38 | 0.132 | 0.444 |
| Ras signaling pathway |  | ecb04014 | 2 | 183 | 0.145 | 0.444 |
| MAPK signaling pathway |  | ecb04010 | 2 | 198 | 0.164 | 0.445 |
| Cardiac muscle contraction |  | ecb04260 | 1 | 50 | 0.169 | 0.451 |
| Prolactin signaling pathway |  | ecb04917 | 1 | 55 | 0.184 | 0.451 |
| Melanoma |  | ecb05218 | 1 | 56 | 0.187 | 0.451 |
